# Disseminated tuberculosis presenting as hemophagocytic lymphohistiocytosis in an immunocompetent adult patient: a case report

**DOI:** 10.1186/s13256-015-0772-8

**Published:** 2015-12-29

**Authors:** P V T M Rathnayake, W K S Kularathne, G C V De Silva, B M S B Athauda, S N N K Nanayakkara, A. Siribaddana, D. Baminiwatte

**Affiliations:** 1 Department of Internal Medicine, General Hospital (Teaching), Kandy, 2000 Sri Lanka; 2 Department of Hematology, General Hospital (Teaching), Kandy, 2000 Sri Lanka; 3 Respiratory Unit 2, General Hospital (Teaching), Kandy, 2000 Sri Lanka; 4 Ophthalmology Unit, General Hospital (Teaching), Kandy, 2000 Sri Lanka

**Keywords:** Hemophagocytic lymphohistiocytosis, Tuberculosis, Choroid tubercles, Immunosuppressants, Endemic, Antituberculous medications, Re-evaluation

## Abstract

**Background:**

Hemophagocytic lymphohistiocytosis is a frequently fatal and likely underdiagnosed disease. It is a rare occurrence in adults and usually secondary to an insult such as viral infections, bacterial infections, autoimmune connective tissue disorders, malignancies and immunocompromised states, in contrast to its childhood counterpart, which is due to a genetic defect but may share some of same genetic etiologies. It is characterized by multisystem inflammation due to unregulated proliferation and infiltration of macrophages and CD8 T cells in the bone marrow, which leads to phagocytosis of red blood cells, platelets, lymphocytes and their precursors.

**Case presentation:**

A 40-year-old Sri Lankan woman presented with a high-grade fever of 2 weeks’ duration and the initial workup, including a thorough clinical examination, and all the investigations, including a septic screen, were normal. On the 18th day of hospital admission, she was found to have yellowish retinal lesions, which were confirmed as choroid tubercles by the consultant eye surgeon. Two days later she became pancytopenic and a bone marrow biopsy confirmed the diagnosis of hemophagocytic lymphohistiocytosis. She was treated with conventional category-1 antituberculous drugs and an initial 2 weeks with high-dose oral dexamethasone. All the choroid tubercles gradually disappeared and she recovered completely without any complications.

**Conclusions:**

In an adult patient with hemophagocytic lymphohistiocytosis, it is pivotal to understand the underlying etiology, as it needs extensive immunosuppression. If this patient had been treated with immunosuppressants without antituberculous medications, it would have been lethal with disseminated or central nervous system tuberculosis. So, in areas where tuberculosis is endemic, if no underlying cause is found, it may be worth considering antituberculous treatment for these patients. Re-evaluation with thorough clinical examination is of utmost importance in any patient with pyrexia of unknown origin as well as in any disease with unusual manifestations.

## Background

Hemophagocytic lymphohistiocytosis (HLH) is a frequently fatal rare disease entity which is due to aggressive proliferation of histiocytes and T lymphocytes in various tissues [1–4]. It leads to phagocytosis of red blood cells, other white blood cells and platelets within bone marrow, spleen and lymph nodes. The pathophysiology of HLH is complex and thought to be due to the lack of perforin-dependant cytotoxicity in natural killer and cytotoxic T lymphocytes. It has a very aggressive course, which is potentially life-threatening and usually affects genetically susceptible children from birth to 18 months [3, 4]. Adult disease is even more rare and considered “secondary HLH” because it is triggered by a secondary insult such as infections, autoimmune connective tissue disorders, immunodeficient states and malignancies, but there may be some genetic predisposition as well [3]. Out of infections, it commonly occurs secondary to viral infections such as Epstein-Barr virus, cytomegalovirus, parvovirus, herpes simplex, varicella-zoster, measles, human herpesvirus 8 and human immunodeficiency virus (HIV) alone or in combination. Also, it can occur with various bacterial infections (Brucella, Gram-negative bacteria, tuberculosis (TB)), parasites (leishmaniasis) and fungal infections. Autoimmune conditions that can act as triggers are systemic lupus erythematosus (SLE), rheumatoid arthritis, Still’s disease, polyarteritis nodosa (PAN), mixed connective tissue disease (MCTD), pulmonary sarcoidosis, systemic sclerosis, Sjogren’s syndrome and drug reaction with eosinophilia and systemic symptoms (DRESS). Other rare associations are immunosuppressed conditions such as in patients with immune deficiencies including X-linked lymphoproliferative disease, common variable immunodeficiency and in renal or liver transplant recipients [5–7].

At the initial stages these patients may mimic common infective illnesses, pyrexia of unknown origin or hepatitis, with clinical findings such as lymphadenopathy and hepatosplenomegaly, but in the latter stages they go into cytopenias, which are potentially life-threatening if not detected in time and treated with aggressive immunosuppressants [6, 8].

There are very few case reports in the world literature regarding TB leading to HLH in immunocompetent patients and this is the first reported case in Sri Lanka, which has high prevalence of TB.

## Case presentation

A 40-year-old Sri Lankan woman from a small village close to Kandy presented with on-and-off high-grade swinging fever that she had had for 2 weeks. It was associated with arthralgia and myalgia but she was otherwise well. There was no long-standing cough, hemoptysis, alteration of bowel habits, loss of weight or urinary symptoms. She was clinically euthyroid and there was no history to suggest connective tissue disorder. Her past medical history was uneventful apart from childhood rheumatic fever, which was treated with oral penicillin until the age of 21 years. An initial clinical evaluation including a fundal examination was normal apart from mild pallor.

Initial investigations revealed a hemoglobin (Hb) level of 10.9 g/dL (mean corpuscular volume (MCV) 84 fL), white blood cell count (WBC) of 13.7 × 10^3^/μL (granulocytes 85.1 %, lymphocytes 8.1, minimum inhibitory dilution 6.8 %), platelets 383 × 10^3^/μL, erythrocyte sedimentation rate (ESR) of 121 mm, C-reactive protein (CRP) of 84.5 mg/dL, aspartate transaminase (AST) of 61.1 U/L, alanine transaminase (ALT) of 63.7 U/L, serum protein of 7.84 g/dL, serum albumin of 4.02 g/dL, serum bilirubin (total) of 5.7 μmol/L, alkaline phosphatase of 210 U/L, gamma-glutamyl transferase of 23 U/L, international normalized ratio (INR) of 1.01, red blood cell count (RBS) of 4.5 mol/L, serum creatinine of 0.8 mg %, serum sodium of 139 mmol/L, and serum potassium of 4.3 mmol/L. Also, a septic screen including three blood cultures and a urine culture, a chest X-ray and electrocardiogram (ECG) were normal. The blood picture revealed moderate rouleaux formation and polymorphonuclear leukocytosis with cytoplasmic vacuolations suggestive of bacterial infection or inflammation with anemia of chronic disease.

**Fig. 1:**
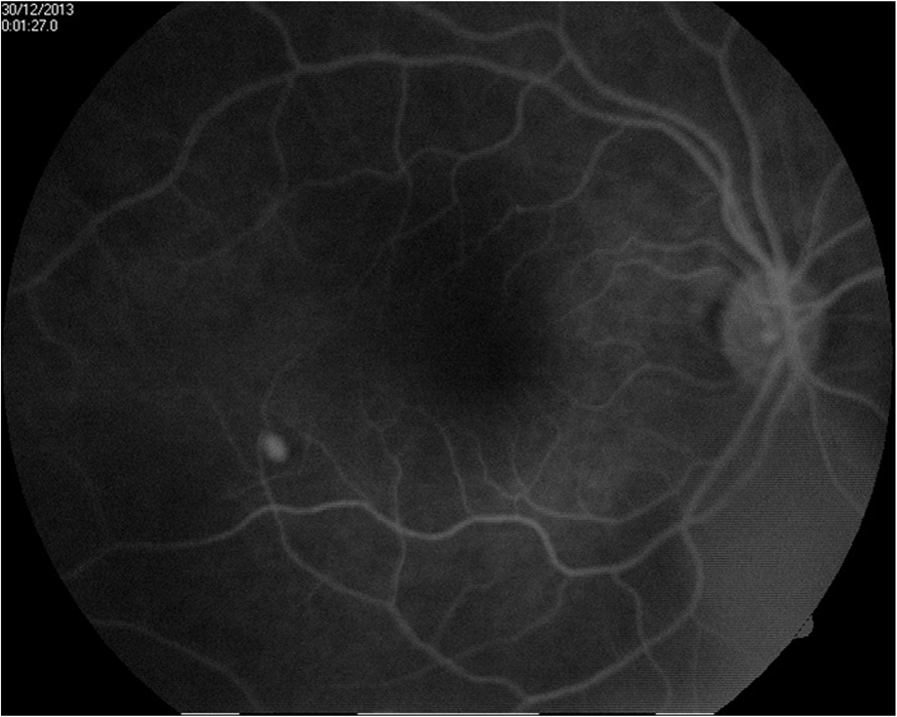
Fluorescein-stained fundal photograph showing a choroid tubercle

She was treated with broad-spectrum antibiotics (ceftriaxone 1 g 12 hourly) and doxycycline for possible rickettsial infection but our patient still continued to have high-grade fever spikes. Hence she underwent second-line investigations including an abdominal ultrasound scan, thick and thin films for malaria, a rheumatoid factor, antinuclear antibody (ANA), and Mantoux test, induced sputum test for acid-fast bacilli, HIV serological test and venereal disease research laboratory (VDRL) test but all results were negative. She was regularly reassessed and on the 18th day of hospital admission found to have yellowish fleshy lesions in her left fundus. They gradually increased in number and extended to the other eye as well. In addition, she was found to have hepatosplenomegaly. Her clinical condition rapidly deteriorated with very high fever spikes (Fig. 1).

**Fig. 2:**
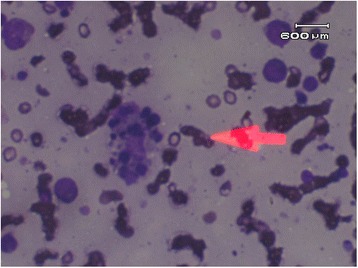
Bone marrow biopsy showing red blood cells engulfed by macrophages (pink arrow)

Repeat investigations revealed a Hb level of 7.7 g/dL (MCV of 84 fL), WBC of 1.7 × 10^3^/ μL (granulocytes 39 %, lymphocytes 31 %, minimum inhibitory dilution 30 %), platelets 49 × 10^3^/μL, ESR of 10 mm, CRP of 93.7 mg/dL, AST of 1705 U/L, ALT of 286 U/L, serum protein of 5.7 g/dL, serum albumin of 3.2 g/dL, serum bilirubin (total) of 25.42 μmol/L, direct bilirubin of 10.1 μmol/L alkaline phosphatase of 724 U/L, gamma-glutamyl transferase of 344.9 U/L, INR of 1.68, and lactate dehydrogenase (LDH) of 1580 U/L. Results of a repeat septic screen were again negative and a chest X-ray did not reveal any significant pathology including miliary mottling. As she was pancytopenic we went ahead with a bone marrow biopsy. It showed increased macrophage activity with engulfed red blood cells, neutrophils and platelets suggestive of hemophagocytic lymphohisteocytosis. There were no visible granulomas. Her serum ferritin value was very high (60,587 ng/mL) with elevated triglycerides (396.3 mg/dL) and her serum fibrinogen level was low (100 mg/dL). A bone marrow culture did not grow acid-fast bacilli (Fig. 2).

She fulfilled criteria for HLH, and because of multiple choroid tubercles in both fundi, TB infection was considered as the trigger [6, 8].

## Discussion

She was treated with high doses of immunosuppressant intravenous methylprednisolone 1 g daily for 3 days followed by a short course of oral dexamethasone for 2 weeks and conventional category 1 anti-TB drugs for 9 months under the supervision of a consultant chest physician. Her deranged liver function test results were attributed to both TB as well as HLH and monitored closely. A liver biopsy was not performed because of her deranged clotting profile. She responded dramatically and her fever settled in crisis with the first dose of intravenous methylprednisolone. The choroid tubercles gradually disappeared after 10 days of anti-TB treatment and her liver function gradually improved without any complications from the anti-TB drugs. She totally recovered from her illness and there has been no relapse up to now, 18 months after her original illness.

## Conclusions

HLH is an uncommon clinical manifestation and, if not suspected early, it can be fatal. In an adult patient especially, it is very important to look for the underlying trigger such as bacterial infections, connective tissue disorders or malignancies because it needs definitive management. As the patient needs high-grade immunosuppression as the treatment for HLH, underlying bacterial infections should be treated promptly [6, 8]. In this case, if she had been treated with immunosuppressants without antituberculous medications, the end result would have been lethal. She was a healthy adult without any underlying immunosuppressive condition, but thorough and repeated clinical examinations clinched the diagnosis of extrapulmonary TB.

Therefore, although we have very sophisticated laboratory investigations, regular reassessments as well as prompt clinical examination are still very important. Also, in areas with high prevalence of TB, it is worth treating HLH patients with anti-TB drugs if there is no secondary cause found because it could be lifesaving.

## Consent

Written informed consent was obtained from the patient for publication of this case report and any accompanying images. A copy of the written consent is available for review by the Editor-in-Chief of this journal.

## Abbreviations

ANA: Antinuclear antibody
ALT: Alanine transaminase
AST: Aspartate transaminase
CRP: C-reactive protein
DRESS: Drug reaction with eosinophilia and systemic symptoms
ECG: Electrocardiogram
ESR: Erythrocyte sedimentation rate
Hb: hemoglobin
HIV: Human immunodeficiency virus
HLH: Hemophagocytic lymphohistiocytosis
INR: International normalized ratio
LDH: Lactate dehydrogenase
MCTD: Mixed connective tissue disease
MCV: Mean corpuscular volume
PAN: Polyarteritis nodosa
RBS: Random blood sugar
SLE: Systemic lupus erythematosus
TB: Tuberculosis
VDRL: Venereal disease research laboratory test
WBC: White blood cell count

## References

[1] Rosado FGN, Kim AS. Hemophagocytic lymphohistiocytosis: an update on diagnosis and pathogenesis. Am J Clin Pathol. 2013;139:713–27. 10.1309/AJCP4ZDKJ4ICOUAT 23690113

[2] Deepak N, Chethan M, Nalini B. Hemophagocytic lymphohistiocytosis masquerading as sepsis. Int J Clin Surg Adv. 2013;1:2–5.

[3] Gunasekera TMR, de Silva S, Vidyatilaka HMS, Faizal MAM. A case of haemophagocytic lymphohistiocytosis (HLH). Sri Lanka J Child Health. 2008;37:98–9.

[4] Filipovich AH. Hemophagocytic lymphohistiocytosis (HLH) and related disorders. Hematology. 2009;1:127–31. 10.1182/asheducation-2009.1.127 20008190

[5] Janka G, zur Stadt U. Familial and acquired hemophagocytic lymphohistiocytosis. Hematology. 2005;1:82–8. 10.1182/asheducation-2005.1.82 16304363

[6] Jordan MB, Allen CE, Weitzman S, Filipovich AH, McClain KL. How I treat hemophagocytic lymphohistiocytosis. Blood. 2011;118:4041–52. 10.1182/blood-2011-03-278127 PMC3204727 21828139

[7] Aouba A, Noguera ME, Clauvel JP, Quint L. Haemophagocytic syndrome associated with Plasmodium vivax infection. Br J Haematol. 2000;108:832–3. 10.1046/j.1365-2141.2000.01968.x 10792290

[8] Hemophagocytic Lymphohistiocytosis Study Group. Treatment protocol of the second international study of HLH. 2014.

